# Observational Study Assessing Demographic, Economic and Clinical Factors Associated with Access and Utilization of Health Care Services of Patients with Multiple Sclerosis under Treatment with Interferon Beta-1b (EXTAVIA)

**DOI:** 10.1371/journal.pone.0113933

**Published:** 2014-11-24

**Authors:** Georgios Hadjigeorgiou, Efthimios Dardiotis, Georgios Tsivgoulis, Triantafyllos Doskas, Damianos Petrou, Nikolaos Makris, Nikolaos Vlaikidis, Thomas Thomaidis, Athanasios Kyritsis, Nikolaos Fakas, Xoulietta Treska, Clementine Karageorgiou, Stefania Sotirli, Christos Giannoulis, Dimitra Papadimitriou, Ioannis Mylonas, Evaggelos Kouremenos, Georgios Vlachos, Dimitrios Georgiopoulos, Despoina Mademtzoglou, Michalis Vikelis, Elias Zintzaras

**Affiliations:** 1 Neurology Department at University Hospital of Larissa, Larissa, Greece; 2 Second Department of Neurology, University of Athens, School of Medicine, “Attikon” University Hospital, Athens, Greece; 3 Department of Neurology, Athens Naval Hospital, Athens, Greece; 4 Outpatient Clinic at Vostanio General Hospital of Mytilini, Mytilini, Greece; 5 Neurology Department at Agios Andreas General University of Patra, Patra, Greece; 6 3rd University Neurology Department at Papanikolaou University Hospital of Thessaloniki, Thessaloniki, Greece; 7 Neurology Department at Greek Red Cross Hospital, Athens, Greece; 8 University Neurology Department at University Hospital of Ioannina, Ioannina, Greece; 9 Neurology Department at 401 Military Hospital of Athens, Athens, Greece; 10 2nd University Neurology Department at AHEPA General University Hospital of Thessaloniki, Thessaloniki, Greece; 11 Neurology Department at Gennimatas General Hospital, Athens, Greece; 12 Private Practice, Athens, Greece; 13 Outpatient Clinic at Interbalcan Medical Center, Thessaloniki, Greece; 14 Neurology Department at 251 Air Force Hospital, Thessaloniki, Greece; 15 Medical Department, Novartis Hellas S.A.C.I., Athens, Greece; 16 BECRO, Pharmaceutical Services, Athens, Greece; 17 Department of Biomathematics, University of Thessaly School of Medicine, Larissa, Greece; 18 The Institute for Clinical Research and Health Policy Studies, Tufts University School of Medicine, Boston, MA, United States of America; Institute Biomedical Research August Pi Sunyer (IDIBAPS) - Hospital Clinic of Barcelona, Spain

## Abstract

Multiple sclerosis (MS) results in an extensive use of the health care system, even within the first years of diagnosis. The effectiveness and accessibility of the health care system may affect patients' quality of life. The aim of the present study was to evaluate the health care resource use of MS patients under interferon beta-1b (EXTAVIA) treatment in Greece, the demographic or clinical factors that may affect this use and also patient satisfaction with the health care system. Structured interviews were conducted for data collection. In total, 204 patients (74.02% females, mean age (SD) 43.58 (11.42) years) were enrolled in the study. Analysis of the reported data revealed that during the previous year patients made extensive use of health services in particular neurologists (71.08% visited neurologists in public hospitals, 66.67% in private offices and 48.53% in insurance institutes) and physiotherapists. However, the majority of the patients (52.45%) chose as their treating doctor private practice neurologists, which may reflect accessibility barriers or low quality health services in the public health system. Patients seemed to be generally satisfied with the received health care, support and information on MS (84.81% were satisfied from the information provided to them). Patients' health status (as denoted by disease duration, disability status and hospitalization needs) and insurance institute were found to influence their visits to neurologists. Good adherence (up to 70.1%) to the study medication was reported. Patients' feedback on currently provided health services could direct these services towards the patients' expectations.

## Introduction

Multiple sclerosis (MS) is a major cause of neurologic impairment in young adults. Acute relapses and chronic disease progression can lead to irreversible lifelong disability. Physical impairment and other MS related symptoms result in an extensive use of the health care system by MS patients, even within the first years of diagnosis [1], [2]. In an economic, social and demographical environment that rapidly changes and variously influences the health system, it is of vital importance to be aware of the burden of MS patients on the health care system in order to confront the requirements and apply novel approaches and services for counteracting the problems. MS has a pronounced impact on health-related quality of life, even from the early stages of the disease. However, the perception of quality of life and health care objectives may differ between clinicians and patients: while doctors mainly focus on physical disability estimates, patients may also be concerned about other aspects of quality of life such as vitality, general and mental health [3] and satisfaction with the health care they are provided with [4], [5]. Health care needs of patients with chronic diseases such as MS are not always obvious and doctors should learn how to detect and assess them in order to deliver optimal care to patients.

There is limited knowledge, especially in Greece, regarding the extent of health services use by MS patients under current immunomodulatory treatments, the inconveniences they may encounter and their satisfaction with these services. The present study was conducted with the objective of estimating the extent of health professional and service use by patients with MS under treatment with interferon beta-1b. In addition, patient satisfaction with health care help/support, the information on MS they received, and treatment compliance were evaluated. We also investigated for possible associations between these objectives and baseline demographic, social and clinical characteristics of the study group.

## Study Design and Population

This was a non-interventional, observational, post-marketing study with retrospective data collection from patients' medical records and direct personal interviews and it was conducted in accordance with the requirements of the European Clinical Trials Directive 2001/20/EC for non-interventional studies.

The study included patients with clinically isolated syndrome (CIS) at high risk of developing clinically definite MS, relapsing remitting MS (RRMS) patients with two or more clinical or imaging relapses within the last two years and secondary progressive MS (SPMS) patients with active disease evidenced by relapses, all of whom were already under treatment with interferon beta-1b (EXTAVIA) for at least one month prior to enrolment according to the drug's indications, the doctors' judgment and the clinical directives on MS treatment [6], [7]. Patients attended public hospitals or doctors' private offices. In total, patients from 21 centers throughout Greece were enrolled in the study. Written informed consent was obtained from all patients prior to their enrollment. The study protocol and the informed consent form were evaluated and approved by the relevant institutional review boards: University Hospital of Larissa, Athens Naval Hospital, Vostanio General Hospital of Mytilini, Agios Andreas General University of Patra, Papanikolaou University Hospital of Thessaloniki, Greek Red Cross Hospital, University Hospital of Ioannina, 401 Military Hospital of Athens, AHEPA General University Hospital of Thessaloniki, Gennimatas General Hospital of Athens, Interbalcan Medical Center of Thessaloniki and 251 Air Force Hospital.

Data collection took place between November 8, 2011 (first patient, first visit) and July 31, 2012 (last patient, last visit). The study was completed in one visit and no formal follow-up was planned in the study centers except for patients who might have presented specific adverse effects (serious or not). A structured questionnaire in suitable Case Report/Record Forms (CRFs) was used for patients' data collection. Participants were interviewed face-to-face by their treating neurologist. Recorded demographic data included age, gender, urban or rural residence, literacy/level of education, occupation and medical insurance. In addition, certain clinical data were recorded, including the date of disease diagnosis, disease duration, disability status (as defined by the Expanded Disability Status Scale, EDSS) hospitalization requirements, visits to one-day clinics and treatment duration. We recorded hospitalizations related or not to MS when patients admitted for MS relapses and complications of the disease or for other medical problems respectively. Furthermore, the number of magnetic resonance imaging (MRI) scans and blood tests performed within the last year was recorded. Health service use by MS patients under interferon beta-1b treatment was recorded based on the nature and frequency of visits to a variety of different health care professionals as well as the frequency of MRI scans and blood tests. The difficulties that these patients encountered in accessing and using health services were directly depicted by the frequency of visits and examinations and by the reimbursement of expenses. Conclusions could further be drawn indirectly, by evaluating patient satisfaction concerning their experience of health service use. Their assessment of the help, support and information on MS received from a number of professionals and groups was evaluated using a four-grade scale questionnaire, ranging from A (excellent/very good) to D (insufficient). Compliance with treatment was identified through specified questions and the frequency of drug omission was recorded. Finally, all information regarding the nature, severity, means of treatment and consequences of adverse events (AEs) and serious AEs, possible pregnancies and their outcome had to be recorded and reported to the Pharmacovigilance service. The study center investigators had to estimate and record the relationship of each SAE to the study drug and appropriately manage it according to current medical practice.

All participating investigators received education on the study protocol, safety reporting requirements and study procedures by the sponsor. During the study, quality control monitoring for possible protocol deviations was conducted. Monitored CRFs were transferred or electronically sent from the sponsor to the clinical research organization (CRO) which was responsible for data entry and database creation.

## Statistical Analysis

Data from categorical and continuous variables were summarized using descriptive statistics and are presented in frequency tables and graphs. In order to determine statistically significant differences within different subgroups over the health care system use, and patient satisfaction with the information and the support they received, the study population was divided into subgroups based on specified baseline characteristics (age, gender, residence, education, employment status, insurance, disability status, disease and treatment duration, hospitalization and visits to one-day clinics) (**table S1**). The most appropriate cut-off points were selected. The value 3 was used as the cut-off of EDSS as it was shown to represent a critical disability milestone in the natural history of MS [8]. For the variables of age, disease and treatment duration the sample medians split were used as the cut-off points. A Chi-square test was used for comparisons between categorical variables within different subgroups of the population, whereas a t-test or one-way ANOVA was used for continuous variables. The Bonferroni correction method for multiple comparisons was applied in the post-hoc tests of the one-way ANOVA.

## Results

In total, 204 patients were included in this study. To date there are relatively limited data available and inconsistencies exist between studies regarding the prevalence rates of MS in Greece. According to the Greek MS societies, the number of MS patients in Greece is estimated to be about 9,500.

Baseline demographic characteristics and clinical data of the study population are summarized in **table 1**. The mean age of the population was 43.58 years, with female predominance (74.02%). Most of the patients had received at least a secondary education (80.39%), lived in major urban centers (60.78%), were employed (56.86%) and had minimal disability (55.88% with EDSS ≤2.5). Regarding treatment duration, most patients had been under interferon beta-1b treatment for one to 12 months (60.78%), while many had been receiving the study drug for one to two years (32.84%).

**Table 1:** Demographic and clinical attendance characteristics of the studied population (n = 204).

**Age**	
Mean	43.58
SD	11.42
Range	19–71
**Sex**	
Male, n (%)	53 (25.98)
Female, n (%)	151 (74.02)
**Disease duration (months)** a	
Mean	89.83
SD	77.31
Range	1–313
**Disability status (according to EDSS)**	
≤2.5, n (%)	114 (55.88)
3.0–5.0, n (%)	69 (33.82)
≥5.5, n (%)	21 (10.29)
**Treatment duration (months)**	
Mean	12.01
SD	9.58
Range	1–60
**Residency**	
Away from urban center, n (%)	63 (30.88)
Athens, n (%)	55 (26.96)
Within Attica prefecture, n (%)	17 (8.33)
Salonika, n (%)	12 (5.88)
Other urban centers, n (%)	57 (27.94)
**Education**	
Secondary, n (%)	112 (54.90)
Higher, n (%)	52 (25.49)
Primary, n (%)	37 (18.14)
No official, n (%)	3 (1.47)
**Employment status**	
Private sector, n (%)	58 (28.43)
Public sector, n (%)	41 (20.10)
Retired, n (%)	23 (11.27)
Retired (due to disability), n (%)	23 (11.27)
Unemployed, n (%)	20 (9.80)
Freelance, n (%)	17 (8.33)
Other, n (%)	22 (10.78)
**Insurance** b	
IKA, n (%)	107 (52.45)
Other public, n (%)	50 (24.51)
OPAD, n (%)	35 (17.16)
OAEE, n (%)	10 (4.90)
Private, n (%)	3 (1.47)

^a^Duration since diagnosis of CIS or clinically definite MS until study enrollment.

^b^IKA: Social Insurance Institute, OPAD: Insurance institute for employees of the public sector, ΟAEE: Insurance institute for freelancers. One patient had both “other public insurance” and “private insurance”.

Results relating to health service use, information regarding patients' medical attendance, hospitalization requirements, need and insurance coverage for MRI and blood tests are presented in **table 2**. The majority of the patients reported attending private practice doctors (52.45%), had undergone MRI (96.57%) and blood test (99.02%) examinations and did not require hospitalization (37.25%) during the last year. Most of the patients (91.18%, 186/204) were not hospitalized due to problems other than MS. It was shown that administrative issues with health services caused an absence from work in 58.89% of employed patients for 10.39±40.42 (mean ± SD) days. Shorter disease duration was associated with more days away from work (p = 0.024, **table S2**).

**Table 2:** Patients' medical attendance, hospitalization and laboratory requirements of the studied population (n = 204).

**Patients' medical attendance**	
Private practice, n(%)	107 (52.45)
Public hospital, n(%)	97 (47.55)
Private hospital, n(%)	0 (0.00)
**Hospitalizations**	
Only visit to one day clinic, n(%)	18 (8.82)
Only hospitalizations, n(%)	39 (19.12)
Neither, n(%)	76 (37.25)
Both, n(%)	71 (34.80)
**Number of admissions** a	
Mean	0.83
SD	1.00
Range	0–6
**Days of admissions**	
Mean	3.05
SD	4.34
Range	0–30
**Number of visits to One-day clinics**	
Mean	1.77
SD	2.98
Range	0–12
**MRI scans**	
**Patients undergone MRI, n (%)**	197 (96.57)
**Number of MRI scans**	
Mean	1.87
SD	1.23
Range	0–8
**MRI scan cost covered by insurance** b	
Yes, n(%)	184 (93.40)
No, n(%)	13 (6.60)
**Blood tests**	
**Patients undergone blood tests, n (%)**	202 (99.02)
**Number of blood tests**	
Mean	2.75
SD	2.13
Range	0–12
**Blood test cost covered by insurance** c	
Yes, n(%)	185 (91.58)
No, n(%)	17 (8.42)

^a^Number of patients' admissions to hospitals to counteract relapses within the last year. In this category, the visits to one-day clinics are not included.

^b^This information was missing in 7 patients.

^c^This information was missing in 2 patients.

The frequency of health care service use related to visits to neurologists, other doctors and health professionals is summarized in **table 3**. The majority of patients visited neurologists in public hospitals (71.08%). However, a considerable proportion of patients referred to neurologists not affiliated to the patients' insurance/neurologists in private clinics (66.67%) or to neurologists affiliated to the patients' insurance institute (48.53%). Of note, almost half of the treated population (42.65%) had visited all these categories of neurologists while 61 patients (29.90%) referred exclusively to one of the three categories. The major baseline demographic and/or clinical data that significantly influenced patients' visits to neurologists seemed to be health status (as denoted by disease duration, disability status and hospitalization needs) and insurance institute (p value<0.05, **table S3**). During the last year, patients also consulted other specialists including internal medicine doctors, orthopedists and ophthalmologists. EDSS score, disease duration, age, hospitalization, education level, place of residence and type of insurance were found to influence the visits to other specialties (p value<0.05, **table S4**). Health service use by our group of MS patients was not limited to doctors but also included visits to other health professionals including physiotherapists, psychologists, social workers, logotherapists and ergotherapists. The cost of almost all visits to these health care professionals was covered by the patients' insurance. Several demographic and clinical characteristics seemed to influence the visits to these health care professionals including age, disease duration, disability status and hospitalization or visits to one-day clinics (p value<0.05, **table S5**).

**Table 3:** Health care service use related to visits to neurologists, other doctors and health professionals.

		Frequency		
	N (%)	mean	SD	range
**Neurologist in public hospitals**	145 (71.08)	2.00	2.54	0–14
**Neurologist in private**	136 (66.67)	1.54	1.73	0–12
**Neurologist affiliated to patient' s insurance**	99 (48.53)	1.14	1.91	0–14
**Internal medicine specialist**	100 (49.02)	1.74	1.06	1–5
**Orthopedist**	40 (19.61)	1.60	1.55	1–10
**Ophthalmologist**	50 (24.51)	1.36	0.66	1–3
**Other doctor specialties**	25 (12.25)	2.40	2.93	1–15
**Any other health care professionals**	78 (38.24)			
Physiotherapist	56 (27.45)	22.46	27.74	1–99
Psychologist	21 (10.29)	3.52	2.98	1–10
Social worker	7 (3.43)	5.00	4.00	1–12
Logotherapist	4 (1.96)	2.25	1.26	1–4
Ergotherapist	4 (1.96)	3.00	2.16	1–6
Other Health care professionals	2 (0.98)			

The patients' opinion on the information and the help/support they received for MS is summarized in **table 4**. In general, the overwhelming majority of the patients (84.81% gave scores A and B) were fairly satisfied with the information provided to them; information was provided mainly by doctors and patient unions and, to a lesser extent, by mass media or by friends and relatives. Patients with a better education and with visits to one-day clinics gave higher scores to the sources of information in general. In addition, their opinion on the information provided to them by friends and relatives was associated with disease and treatment duration as well as with previous experience of hospitalization or visits to one-day clinics (p<0.05, **table S6**).

**Table 4:** Patient evaluation of the received information and help/support on multiple sclerosis.

	n(%)^a^			
	A (excellent-very good)	B (good-satisfactory)	C (medium)	D (insufficient)
**Source of information on MS**				
Independent to the source	42 (20.59)	131 (64.22)	27 (13.24)	4 (1.96)
Doctor	92 (45.32)	106 (52.22)	5 (2.46)	0 (0.00)
Print media	3 (1.49)	79 (39.30)	90 (44.78)	29 (14.43)
Electronic media	17 (8.42)	96 (47.52)	68 (33.66)	21 (10.40)
Patient union^b^	8 (4.55)	89 (50.57)	42 (23.86)	37 (21.02)
Friends/relatives	6 (3.05)	59 (29.95)	74 (37.56)	58 (29.44)
Other	0 (0.00)	5 (35.71)	5 (35.71)	4 (28.57)
**Received help/support on MS**				
Doctors and health professionals	85 (41.67)	98 (48.04)	18 (8.82)	3 (1.47)
Insurance institutes on MS-related problems	26 (12.75)	86 (42.16)	75 (36.76)	17 (8.33)
Employer's behavior at work place for MS-related problems	27 (18.00)	61 (40.67)	45 (30.00)	17 (11.33)
Procedure of treatment administration by the insurance institute	48 (23.53)	92 (45.10)	56 (27.45)	8 (3.92)
Behavior/support by patient support programs^c^	25 (16.56)	72 (47.68)	40 (26.49)	14 (9.27)

^a^Any patient with missing values was excluded from analysis due to lack of information retrieval by the corresponding source.

^b^Absence of answer in the relevant field of the CRF was considered as no participation in patient union. In total, 86.27% of the study population reported participation in MS unions.

^c^Absence of answer in the relevant field of the CRF was considered as no participation in patient support program. In total, 74.02% of the study population reported participation in support programs.

Regarding the patients' opinion on help and support received, they seemed to be highly satisfied with the help and support they received from their doctors and other health professionals and with the behavior of patient support programs and of their employers. Furthermore, they rated the support of their insurance institutes on MS-related problems and the medication administration as above average. The assessment of patient support programs was significantly related to the patients' education level as well as disease and treatment duration. The patients' perceived help/support from their insurance or employers were significantly influenced by the type of insurance or level of education, respectively (p<0.05, **table S7**).

The evaluation of treatment compliance was included among the objectives of the present study and was estimated by the number of times the patient omitted the treatment within the last month for any reason. Statistical analysis showed that only 0.59±1.13 (mean ± SD) doses were omitted within the last month, while 70.10% of the patients did not omit any doses at all. Patients with higher EDSS scores, longer disease and treatment duration were more prone to omit doses (p value<0.05, **table S8, table S9**).

Finally, with regard to AEs, flu-like symptoms (33.33%, 6/18), elevated body temperature (22.22%, 4/18) and fatigue (22.22%, 4/18) were the most common AEs. The majority of them were mild in severity (88.89%, 16/18) and nearly all were considered to be related to the study drug (94.44%, 17/18). Pharmaceutical treatment was administered for 44.44% (8/18) of AEs whereas no action was taken in 50.00% (9/18) of cases. No serious adverse events or cases of pregnancy were reported.

We tested for possible association between the reported AEs and the omission of interferon beta-1b injections. Treatment omission was recorded for 26.32% (5/19) of patients with AEs and 30.27% (56/185) of patients without AEs. No statistically significant association was observed between treatment compliance and the reported AEs (Chi-square: 0.129, p-value: 0.720).

## Discussion

MS has a pronounced impact on health-related quality of life, even during the first stages of the disease. The ability to walk, as well as fatigue, depression, pain, bladder and mental problems are only some of the challenging aspects of an MS patient's life. All of these factors contribute to extensive health service use. Assessing the accessibility and effectiveness of and the burden on the health care system may be of vital importance in order to meet patients' requirements and to apply novel approaches. In the present study, using a structured interviewing approach, we analyzed patients' health service use and we investigated possible influence by baseline demographic, social and clinical characteristics of the study group.

The health care system in Greece, as a consequence of economic crisis and the austerity measures, was recently reformed in 2011 [9]. The government, in order to reduce administrative costs and achieve equal accessibility of all citizens to health care, merged all social insurance funds into one unified fund, the EOPYY, which covers almost all of the Greek population. The reform aimed to maintain universal access of all citizens to health care services. In addition, public insurance plans were unified across the country. For example, for multiple sclerosis, the public insurance plan includes, among the others, full coverage of medical attendance by neurologists in public hospitals or those affiliated to the insurance, full coverage of the immunomodulatory treatment and partial coverage of paramedical examinations (for example MRIs or blood tests). However, the austerity policies, directly or not, shifted several costs to patients leading to a reduction in health care access [10]. In addition, the income shrinkage of the population led to increased demands of public health care services, which, however, were not always able to be met. Moreover, the number of the medical professionals, who are required to provide high-level health services, is critical in several public health care institutions. As a result of the increased demand of health services and the limited number of public health professionals, patients often face lengthy waits, sometimes up to one month until they get examined in public institutions. Considering this health situation in Greece, our study provides some valuable clues as to the health care resource use of MS patients.

Regarding health care service use, our study showed that MS patients frequently referred to neurologists of either the public or the private sector or those provided by their insurance. However, a substantial amount of patients, although they can be attended by neurologists in public hospitals or affiliated to the insurance without any cost, prefer to visit private neurologists for their problems or choose private neurologists as their treating physicians. This choice may reveal accessibility difficulties, low quality of the provided care or may reflect serious underlying structural problems and inefficiencies concerning organization, funding and delivery of services [11]. The type of insurance and the health status of patients seemed to significantly affect their visits to neurologists. Of note, many patients visited all three categories of neurologists maybe in an attempt to ensure the best attention and treatment decisions. In addition, patients with shorter disease duration and lower disability score were found to entrust their care to non-private neurologists, whereas patients with longer disease duration or higher disability scores tended to prefer private neurologists. Previous studies have reported quite similar findings regarding the frequency and number of annual visits to neurologists [1], [12] or the baseline characteristics that affect visits to neurologists [12], [13]. Half of the patients visited an internal medicine specialist during the last year. However, this is lower than would be expected from previous studies [1], [14], [15] probably as a result of the structure of the health care system in Greece where a patient can refer directly to a neurologist without having first to visit a general practitioner or an internal medicine doctor. In our study, patients' decisions to visit doctors other than neurologists were found to be associated with their level of education, residence and score on the EDSS scale of disability status. Previous reports have also noticed higher visit rates to doctors by patients with a higher severity of MS [13]. On the other hand, our results on the effect of residence on health service access are opposite to what was expected from previous studies, since MS patients living in rural areas were shown to have more barriers to care compared with those living in urban areas [16]. Approximately one third of our study population also visited physiotherapists and, less frequently, other health professionals. As expected [4], MS patients of advanced age, longer disease duration and higher EDSS score visited a physiotherapist more often. The majority of the visits to other health care professionals were reimbursed by the patients' insurance. Previous studies have also shown that MS patients, especially those with advanced disease, seek the help of physiotherapists relatively often [13], [17].

The present study also aimed to estimate the difficulties that patients encountered in accessing these services and their satisfaction with them, in order to ensure improved responsiveness to patients' needs and preferences as well as to deliver optimized care that meets perceived needs. Our study showed that patients were satisfied with the information they received in general and they further appreciated the information provided to them by their doctors (97.54%). These high rates of satisfaction compared with other studies [4], [18] might have been affected by the presence of the physician in patients' interviews. Patients with frequent visits and a better educational background, possibly resulting in better understanding, were more satisfied with the information provided to them. A noteworthy and encouraging finding is that patients were content with the support they received from health care professionals, institutes, employers and support programs. Previous studies in different ethnic groups revealed various levels of satisfaction of MS patients with the care and support they received [4], [18], [19].

The last objective of the present study was the evaluation of treatment compliance. It was demonstrated that only 0.59±1.13 doses were omitted during the previous month, while 70.10% of patients did not omit any doses at all. In previous MS studies, rates of non-adherence to interferon beta-1b i.e., missing one or more injections, have ranged between 16–51% [19]–[23], quite similar to our findings. However, considerably fewer prescribed doses were missed by patients in the present study than in others [20]. The main baseline characteristics that significantly affected treatment compliance in our study were disability status, disease duration and treatment duration, having patients with disease of longer duration or greater severity being more prone to omit a dose. Previous studies have identified the age at diagnosis, disease duration [21], [22] and therapy duration [19] as significant factors for therapy adherence. Another study [24], investigating factors that influence discontinuation of immunomodulatory treatment in MS indicated EDSS score as the main factor that predicted interruption of therapy. Further studies [25] indicated the importance of patient education in avoiding treatment interruption and a similar tendency was implied by the present study, although statistical significance was not reached (p = 0.12 table S8).

Finally, the presence of AEs was recorded, as it is widely accepted that observational post-marketing studies constitute an important part of the evaluation of tolerance and safety of new substances and formulations, especially when wide and long-term use is expected, as in the case of MS treatment. In the present study, and in accordance with previous long-term follow-up studies [26], [27], interferon beta-1b was associated with a favorable tolerability and safety profile and no unexpected or serious AEs were recorded.

However, our study has some limitations. This is an observational analysis with retrospective data collection restricted in a subset of the global MS population and more precisely, only patients from a relatively small country that were under a specific MS treatment were included in this study. In addition, some important questions such as the cost of visits to private doctors, the productivity loss or the impact of the socioeconomic status on the health care use that may help health authorities to design health care reforms were not addressed in the present study, although our previous experience has shown that patients are generally negative to questions concerning economic status [28]. Also, no well-defined or validated questionnaires such as Euro-QoL were used in our study. Moreover, it was beyond the scope of this study to consider a “healthy comparison cohort” for analyses and, thus, it is only possible to estimate the absolute and not the relative burden of MS patients on the health system. Nevertheless, the present results are not to be underestimated, since they record the health service use habits of a considerable sample of the Greek MS population across a great number of medical centers, avoiding a client-centered perspective. Consequently, these results could be representative for different structures in the health care system. Furthermore, in most cases they reflect the previously described tendencies of MS patients and no strong disagreements with the available literature were noted. Although this survey was not tested for reliability/validity and, thus, comparisons with other studies may not be well-founded, this was also the case in previous studies with similar endpoints. Besides, this study focused on an endpoint that is recently emerging as an important factor for the improvement of long-term therapeutic outcomes in chronic illness, namely the consideration of patient views on health care and patient empowerment. It is essential to evaluate patients' feedback and modify health services based on the viewpoint of those they serve. In the future, it would be interesting to assess patients' opinion on further aspects, including, but not limited to, involvement in health care dimensions, preferences on medical staff, care organization, perceived utility of treatment and access to medical centers. Regarding treatment compliance, it is of note that more adherent patients were reported compared with some previous studies, probably indicating the patient satisfaction with the administered treatment or as a result of the active involvement of health providers in convincing the patients about the importance of immunomodulatory treatment, in implementing various supportive elements or devices or effectively counteracting AEs, tasks that are critical to achieving optimal therapeutic adherence. Finally, the study medication seemed to be well-tolerated and there were no reports of AEs, the nature or severity of which has not been previously described.

As an overall conclusion regarding the study objectives that were relevant to health service use, it could be claimed that patients made extensive use of the services of neurologists and physiotherapists and, to a lesser extent, of internal medicine specialists. However, many patients prefered to visit private neurologists or chose private neurologists as their treating physicians which may reflect accessibility barriers, low quality health services in the public health system or other serious structural health care problems. Hence, this must be taken into account by authorities and public health institutions in order to undertake action to meet the increasing needs and improve the quality of health services.

## Supporting Information

Table S1 **Baseline demographic and clinical characteristics used to define subgroups of the treated population.**
(DOCX)

Table S2 **Results of t-tests or one-way ANOVA for estimation of association of the days that the patient had to take off work for administrative issues with his/her insurance institute or other health services with baseline demographic and clinical characteristics of the treated.**
(DOCX)

Table S3 **Results of Chi square tests for estimation of association of neurologist visiting with baseline demographic and clinical characteristics of the treated population.**
(DOCX)

Table S4 **Results of Chi square tests for estimation of association between visiting doctors of other than neurology specialties and baseline demographic and clinical characteristics of the treated population.**
(DOCX)

Table S5 **Results of Chi square tests for estimation of association of health professional visiting with baseline demographic and clinical characteristics of the treated population.** The p-value is presented.
(DOCX)

Table S6 **Results of Chi square tests for estimation of association of patient evaluation on the utility of the received information on multiple sclerosis with baseline demographic and clinical characteristics of the treated population.** The p-value is given.
(DOCX)

Table S7 **Results of Chi square tests for estimation of association of patient evaluation on the support/behavior of a number of professionals and groups with baseline demographic and clinical characteristics of the treated population.** The p-value is given.
(DOCX)

Table S8 **Results of t-tests or one-way ANOVA for estimation of association of treatment omission times with baseline demographic and clinical characteristics of the treated population.**
(DOCX)

Table S9 **Results of Chi square tests for estimation of association of treatment omission with baseline demographic and clinical characteristics of the treated population.**
(DOCX)
