# Correction: Observational study assessing demographic, economic and clinical factors associated with access and utilization of health care services of patients with multiple sclerosis under treatment with interferon beta-1b (EXTAVIA)

**DOI:** 10.1371/journal.pone.0140308

**Published:** 2015-10-06

**Authors:** Georgios Hadjigeorgiou, Efthimios Dardiotis, Georgios Tsivgoulis, Triantafyllos Doskas, Damianos Petrou, Nikolaos Makris, Nikolaos Vlaikidis, Thomas Thomaidis, Athanasios Kyritsis, Nikolaos Fakas, Xoulietta Treska, Clementine Karageorgiou, Stefania Sotirli, Christos Giannoulis, Dimitra Papadimitriou, Ioannis Mylonas, Evaggelos Kouremenos, George S. Vlachos, Dimitrios Georgiopoulos, Despoina Mademtzoglou, Michalis Vikelis, Elias Zintzaras

The eighteenth author’s name is incorrectly spelled. The correct name is George S. Vlachos.
