# Supplementary material for: Observational Study Assessing Demographic, Economic and Clinical Factors Associated with Access and Utilization of Health Care Services of Patients with Multiple Sclerosis under Treatment with Interferon Beta-1b (EXTAVIA)
Source: PLoS One. 2014 Nov 24;9(11):e113933. doi: 10.1371/journal.pone.0113933 (PMC4242657; doi:10.1371/journal.pone.0113933)
Supplement: Table S1 — Baseline demographic and clinical characteristics used to define subgroups of the treated population. (DOCX) [file pone.0113933.s001.docx]

| **Table S1:** Baseline demographic and clinical characteristics used to define subgroups of the treated population | |
| --- | --- |
| **Characteristic** | **Subgroups** |
| Age^a^ | old VS young |
| Gender | male VS female |
| Residence | urban centers VS away from urban centers |
| Education | primary/no official VS secondary VS higher |
| Employment status | working VS not working |
| Insurance^b^ | IKA/OAEE VS OPAD/other public insurance institute |
| Disease duration^c^ | long VS short |
| Disability status (EDSS) | ≤ 2.5 VS ≥ 3.0 |
| Hospitalization | yes VS no |
| Visit to one-day clinic | yes VS no |
| Treatment duration^d^ | long VS short |

^a^ Young those <43 years old

^b^ The patients were roughly divided to those receiving insurance due to working in the public sector and to those whose insurance was due to working in the private sector or in freelance. IKA: Social Insurance Institute, OPAD: Insurance institute for employees of the public sector in Greece, ΟΑΕΕ: Insurance institute for freelancers in Greece

^c^ Short duration of disease <74.5 months

^d^ Short duration of treatment < 9 months
