# Supplementary material for: Observational Study Assessing Demographic, Economic and Clinical Factors Associated with Access and Utilization of Health Care Services of Patients with Multiple Sclerosis under Treatment with Interferon Beta-1b (EXTAVIA)
Source: PLoS One. 2014 Nov 24;9(11):e113933. doi: 10.1371/journal.pone.0113933 (PMC4242657; doi:10.1371/journal.pone.0113933)
Supplement: Table S2 — Results of t-tests or one-way ANOVA for estimation of association of the days that the patient had to take off work for administrative issues with his/her insurance institute or other health services with baseline demographic and clinical characteristics of the treated. (DOCX) [file pone.0113933.s002.docx]

| **Table S2:** Results of t-tests or one-way ANOVA for estimation of association of the days that the patient had to take off work for administrative issues with his/her insurance institute or other health services with baseline demographic and clinical characteristics of the treated | | | |
| --- | --- | --- | --- |
|  | **Days off work (mean ± SD)** | **statistic ^a^** | **p-value** |
| **Characteristic** |  |  |  |
| **Age**  old  young | 9.98 ± 40.441  10.77 ± 40.612 | -0.130 | 0.897 |
| **Gender**  male  female | 13.48 ± 54.069  9.26 ± 34.280 | 0.618 | 0.538 |
| **Residence**  urban centers  away from urban centers | 10.65 ± 36.264  9.84 ± 48.298 | -0.125 | 0.901 |
| **Education**  primary/no official  secondary  higher | 17.47 ± 66.163  9.70 ± 37.528  7.04 ± 17.124 | 0.664 | 0.516 |
| **Insurance**  IKA/OAEE  OPAD/other public | 7.00 ± 16.845  14.68 ± 57.746 | -1.265 | 0.208 |
| **Disease duration**  long  short | 3.41 ± 5.923  16.99 ± 55.424 | -2.272 | **0.024** |
| **Disability status (EDSS)**  ≤ 2.5  ≥ 3.0 | 13.10 ± 51.032  6.64 ± 16.741 | 1.055 | 0.293 |
| **Hospitalization**  yes  no | 15.90 ± 54.457  4.43 ± 11.924 | 1.911 | 0.058 |
| **Visit to one-day clinic**  yes  no | 14.86 ± 44.843  7.24 ± 36.882 | 1.245 | 0.215 |
| **Treatment duration**  long  short | 10.00 ± 41.493  10.75 ± 39.621 | -0.124 | 0.901 |

^a^ t in case of t-test, F in case of one-way ANOVA
