# Supplementary material for: Observational Study Assessing Demographic, Economic and Clinical Factors Associated with Access and Utilization of Health Care Services of Patients with Multiple Sclerosis under Treatment with Interferon Beta-1b (EXTAVIA)
Source: PLoS One. 2014 Nov 24;9(11):e113933. doi: 10.1371/journal.pone.0113933 (PMC4242657; doi:10.1371/journal.pone.0113933)
Supplement: Table S3 — Results of Chi square tests for estimation of association of neurologist visiting with baseline demographic and clinical characteristics of the treated population. (DOCX) [file pone.0113933.s003.docx]

| **Table S3:** Results of Chi square tests for estimation of association of neurologist visiting with baseline demographic and clinical characteristics of the treated population | | | | | | | | |
| --- | --- | --- | --- | --- | --- | --- | --- | --- |
|  | **PRIVATE ^a^** | | **PUBLIC ^a^** | | **INSURANCE ^a^** | | **PRIVATE vs PUBLIC/INSURANCE vs ALL ^a^** | |
|  | **Chi-square** | **p-value** | **Chi-square** | **p-value** | **Chi-square** | **p-value** | **Chi-square** | **p-value** |
| **Characteristic** |  |  |  |  |  |  |  |  |
| **Age** (old VS young) | 2.834 | 0.092 | 7.369 | **0.007** | 1.265 | 0.261 | 7.406 | **0.025** |
| **Gender** (male VS female) | 0.204 | 0.652 | 0.056 | 0.813 | 3.340 | 0.068 | 1.046 | 0.593 |
| **Residence** (urban centers VS away from urban centers) | 0.000 | 1.000 | 1.159 | 0.282 | 2.707 | 0.100 | 6.610 | **0.037** |
| **Education** (primary/no official VS secondary VS higher) | 4.573 | 0.102 | 1.040 | 0.595 | 5.538 | 0.063 | 5.536 | 0.237 |
| **Employment status** (working VS not working) | 0.661 | 0.416 | 3.803 | 0.051 | 10.632 | **0.001** | 10.106 | **0.006** |
| **Insurance** (IKA/OAEE VS OPAD/other public) | 5.772 | **0.016** | 0.069 | 0.794 | 6.821 | **0.009** | 11.372 | **0.003** |
| **Disease duration** (long VS short) | 14.158 | **0.000** | 2.188 | 0.139 | 0.076 | 0.782 | 13.564 | **0.001** |
| **Disability status (EDSS)** (≤ 2.5 VS ≥ 3.0) | 7.247 | **0.007** | 0.376 | 0.540 | 0.036 | 0.849 | 6.922 | **0.031** |
| **Hospitalization** (yes VS no) | 1.931 | 0.165 | 20.217 | **0.000** | 2.054 | 0.152 | 11.408 | **0.003** |
| **Visit to one-day** clinic (yes VS no) | 11.551 | **0.001** | 0.584 | 0.445 | 8.478 | **0.004** | 17.200 | **0.000** |
| **Treatment duration** (long VS short) | 2.838 | 0.092 | 12.981 | **0.000** | 4.353 | **0.037** | 5.225 | 0.733 |

^a^ PRIVATE: the patients were divided to a group that had visited private doctors not affiliated to their insurance institute or doctors in private clinic and to a group that had not visit such neurologists.

PUBLIC: the patient subgroups were defined according to the patient having or not having visited neurologists in public hospitals

INSURANCE: the patients’ subgroups were allocated to two subgroups with the criterion of having or not having visited doctors affiliated to their insurance institute.

PRIVATE VS PUBLIC/INSURANCE VS ALL: the patients were categorized to those that had visited doctors exclusively in private and to those that had not (i.e. in public/affiliated to insurance or in private/in public or in private/affiliated to insurance) and to those that had visited all possible neurologists (i.e. in private, in public and affiliated to insurance)
