# Supplementary material for: Observational Study Assessing Demographic, Economic and Clinical Factors Associated with Access and Utilization of Health Care Services of Patients with Multiple Sclerosis under Treatment with Interferon Beta-1b (EXTAVIA)
Source: PLoS One. 2014 Nov 24;9(11):e113933. doi: 10.1371/journal.pone.0113933 (PMC4242657; doi:10.1371/journal.pone.0113933)
Supplement: Table S4 — Results of Chi square tests for estimation of association between visiting doctors of other than neurology specialties and baseline demographic and clinical characteristics of the treated population. (DOCX) [file pone.0113933.s004.docx]

| **Table S4:** Results of Chi square tests for estimation of association between visiting doctors of other than neurology specialties and baseline demographic and clinical characteristics of the treated population | | | | | | | | | | |
| --- | --- | --- | --- | --- | --- | --- | --- | --- | --- | --- |
|  | **internal medicine specialist ^a^** | | **orthopedist ^a^** | | **ophthalmologist ^a^** | | **other ^a^** | | **all VS none ^a^** | |
|  | **Chi-square** | **p-value** | **Chi-square** | **p-value** | **Chi-square** | **p-value** | **Chi-square** | **p-value** | **Chi-square** | **p-value** |
| **Characteristic** |  |  |  |  |  |  |  |  |  |  |
| **Age** (old VS young) | 0.956 | 0.328 | 2.190 | 0.139 | 4.836 | **0.028** | 2.080 | 0.149 | 0.006 | 0.939 |
| **Gender** (male VS female) | 0.906 | 0.341 | 0.925 | 0.336 | 0.556 | 0.456 | 0.060 | 0.806 | 0.201 | 0.654 |
| **Residence** (urban centers VS away from urban centers) | 4.656 | **0.031** | 0.395 | 0.530 | 0.039 | 0.844 | 0.111 | 0.739 | 6.482 | **0.011** |
| **Education** (primary/no official VS secondary VS higher) | 10.664 | **0.005** | 6.045 | **0.049** | 3.891 | 0.143 | 5.522 | 0.063 | 6.660 | **0.036** |
| **Employment status** (working VS not working) | 2.710 | 0.100 | 1.596 | 0.206 | 0.015 | 0.904 | 0.334 | 0.563 | 0.658 | 0.417 |
| **Insurance** (IKA/OAEE VS OPAD/other public) | 10.879 | **0.001** | 0.000 | 0.983 | 0.190 | 0.663 | 0.334 | 0.563 | 1.203 | 0.273 |
| **Disease duration** (long VS short) | 8.635 | **0.003** | 2.190 | 0.139 | 3.510 | 0.061 | 3.494 | 0.062 | 0.157 | 0.692 |
| **Disability status (EDSS)** (≤ 2.5 VS ≥ 3.0) | 13.204 | **0.000** | 5.091 | **0.024** | 0.095 | 0.758 | 1.697 | 0.193 | 4.212 | **0.040** |
| **Hospitalization** (yes VS no) | 0.162 | 0.688 | 2.676 | 0.102 | 9.178 | **0.002** | 0.023 | 0.878 | 1.128 | 0.288 |
| **Visit to one-day clinic** (yes VS no) | 2.749 | 0.097 | 2.855 | 0.091 | 0.221 | 0.638 | 0.275 | 0.600 | 0.852 | 0.356 |
| **Treatment duration** (long VS short) | 1.934 | 0.164 | 2.214 | 0.137 | 1.715 | 0.190 | 1.633 | 0.201 | 0.242 | 0.623 |

^a^ For the first, second, third and fourth column sets the patients were categorized to those that had visited the pathologist, orthopedist, ophthalmologist, other doctor respectively and to those that did not visit each doctor. The subgroups of the last column set were: i) patients that visited any of the above doctors, ii) patients that visited no doctor
