# Supplementary material for: Observational Study Assessing Demographic, Economic and Clinical Factors Associated with Access and Utilization of Health Care Services of Patients with Multiple Sclerosis under Treatment with Interferon Beta-1b (EXTAVIA)
Source: PLoS One. 2014 Nov 24;9(11):e113933. doi: 10.1371/journal.pone.0113933 (PMC4242657; doi:10.1371/journal.pone.0113933)
Supplement: Table S5 — Results of Chi square tests for estimation of association of health professional visiting with baseline demographic and clinical characteristics of the treated population. The p-value is presented. (DOCX) [file pone.0113933.s005.docx]

| **Table S5:** Results of Chi square tests for estimation of association of health professional visiting with baseline demographic and clinical characteristics of the treated population. The p-value is presented | | | | | | | |
| --- | --- | --- | --- | --- | --- | --- | --- |
|  | **Social worker** | **Psychologist** | **Ergotherapist** | **Physiotherapist** | **Logotherapist** | **Other** | **Any VS none** |
| **Characteristic** |  |  |  |  |  |  |  |
| **Age** (old VS young) | 0.681 | 0.269 | 0.322 | **0.000** | 0.984 | 0.989 | **0.047** |
| **Gender** (male VS female) | 0.111 | 0.069 | 0.964 | 0.266 | 0.231 | 0.436 | 0.743 |
| **Residence** (urban centers VS away from urban centers) | 0.485 | 0.215 | 0.797 | 0.506 | 0.177 | 0.556 | 0.945 |
| **Education** (primary/no official VS secondary VS higher) | 0.764 | 0.077 | 0.960 | 0.322 | 0.960 | 0.443 | 0.054 |
| **Employment status** (working VS not working) | 0.177 | 0.682 | 0.640 | **0.025** | 0.133 | 0.074 | 0.124 |
| **Insurance** (IKA/OAEE VS OPAD/other public) | 0.991 | 0.984 | 0.471 | 0.637 | 0.764 | 0.220 | 0.734 |
| **Disease duration** (long VS short) | 0.720 | 0.520 | **0.041** | **0.000** | **0.041** | 0.151 | **0.002** |
| **Disability status (EDSS)** (≤ 2.5 VS ≥ 3.0) | 0.946 | 0.421 | 0.209 | **0.000** | **0.023** | 0.866 | **0.000** |
| **Hospitalization** (yes VS no) | 0.081 | **0.027** | 0.059 | 0.333 | 0.059 | 0.922 | **0.010** |
| **Visit to one-day clinic** (yes VS no) | **0.021** | **0.022** | 0.780 | 0.291 | 0.459 | 0.844 | **0.004** |
| **Treatment duration** (long VS short) | **0.010** | 0.616 | 0.937 | 0.811 | 0.937 | 0.956 | 0.775 |

For the columns “social worker”, “psychologist”, “ergotherapist”, “logotherapist”, “other health professional” the patients were categorized to: i) those that visited the relevant health professional, ii) those that did not visit that specific health professional. In the case of the column referring to “physiotherapist” the patients were grouped to: i) those that did not visit physiotherapist, ii) those that visited physiotherapist 1-10 times, iii) those that visited physiotherapist at least 11 times. The subgroups of the last column were: i) patients that visited any of the above health professionals, ii) patients that visited no health professional
