# Supplementary material for: Observational Study Assessing Demographic, Economic and Clinical Factors Associated with Access and Utilization of Health Care Services of Patients with Multiple Sclerosis under Treatment with Interferon Beta-1b (EXTAVIA)
Source: PLoS One. 2014 Nov 24;9(11):e113933. doi: 10.1371/journal.pone.0113933 (PMC4242657; doi:10.1371/journal.pone.0113933)
Supplement: Table S6 — Results of Chi square tests for estimation of association of patient evaluation on the utility of the received information on multiple sclerosis with baseline demographic and clinical characteristics of the treated population. The p-value is given. (DOCX) [file pone.0113933.s006.docx]

| **Table S6:** Results of Chi square tests for estimation of association of patient evaluation on the utility of the received information on multiple sclerosis with baseline demographic and clinical characteristics of the treated population. The p-value is given | | | | | | | |
| --- | --- | --- | --- | --- | --- | --- | --- |
|  | **Received information on MS^a^** | | | | | | |
|  | **Independently of source** | **Doctor** | **Print media** | **Electronic media** | **Patient union** | **Friends/ relatives** | **Other source** |
| **Characteristic** |  |  |  |  |  |  |  |
| **Age** (old VS young) | 0.799 | 0.627 | 0.730 | **0.011** | 0.555 | 0.392 | 0.577 |
| **Gender** (male VS female) | 0.387 | 0.474 | 0.902 | 0.280 | 0.641 | 0.753 | 0.052 |
| **Residence** (urban centers VS away from urban centers) | 0.547 | 0.604 | 0.972 | 0.704 | 0.647 | 0.368 | 0.207 |
| **Education** (primary/no official VS secondary VS higher) | **0.031** | 0.489 | 0.835 | 0.068 | 0.875 | 0.513 | 0.326 |
| **Employment status** (working VS not working) | 0.110 | 0.942 | 0.718 | 0.053 | 0.441 | 0.633 | 0.158 |
| **Insurance** (IKA/OAEE VS OPAD/other public) | 0.483 | 0.896 | 0.236 | 0.703 | 0.541 | 0.239 | 0.577 |
| **Disease duration** (long VS short) | 0.519 | 0.164 | 0.096 | 0.119 | 0.231 | **0.050** | 0.577 |
| **Disability status (EDSS)** (≤ 2.5 VS ≥ 3.0) | 0.192 | 0.099 | 0.343 | **0.005** | 0.633 | 0.875 | 0.872 |
| **Hospitalization** (yes VS no) | 0.074 | 0.132 | 0.181 | 0.309 | 0.126 | **0.039** | 0.480 |
| **Visit to one-day clinic** (yes VS no) | **0.012** | **0.048** | 0.140 | 0.825 | 0.763 | **0.049** | 0.649 |
| **Treatment duration** (long VS short) | 0.129 | 0.595 | 0.682 | 0.172 | 0.845 | **0.006** | 0.334 |

^a^The patients were categorized to those finding the received information excellent-very good or good-satisfactory and to those estimating the information as medium or insufficient.
