# Supplementary material for: Observational Study Assessing Demographic, Economic and Clinical Factors Associated with Access and Utilization of Health Care Services of Patients with Multiple Sclerosis under Treatment with Interferon Beta-1b (EXTAVIA)
Source: PLoS One. 2014 Nov 24;9(11):e113933. doi: 10.1371/journal.pone.0113933 (PMC4242657; doi:10.1371/journal.pone.0113933)
Supplement: Table S7 — Results of Chi square tests for estimation of association of patient evaluation on the support/behavior of a number of professionals and groups with baseline demographic and clinical characteristics of the treated population. The p-value is given. (DOCX) [file pone.0113933.s007.docx]

| **Table S7:** Results of Chi square tests for estimation of association of patient evaluation on the support/behavior of a number of professionals and groups with baseline demographic and clinical characteristics of the treated population. The p-value is given | | | | | |
| --- | --- | --- | --- | --- | --- |
|  | **Received help/support on MS^a^** | | | | |
|  | **Help/support of doctors and health professionals** | **Help/support of insurance institutes on MS-related problems** | **Employer’s behavior at work place for MS-related problems** | **Procedure of drug administration by the insurance institute** | **Behavior/support by patient support programs** |
| **Characteristic** |  |  |  |  |  |
| **Age** (old VS young) | 0.781 | 0.877 | 0.087 | 0.582 | 0.971 |
| **Gender** (male VS female) | 0.417 | 0.189 | 0.382 | 0.688 | 0.372 |
| **Residence** (urban centers VS away from urban centers) | 0.215 | 0.463 | 0.565 | 0.781 | 0.581 |
| **Education** (primary/no official VS secondary VS higher) | 0.184 | 0.943 | **0.036** | 0.137 | **0.044** |
| **Employment status** (working VS not working) | 0.138 | 0.692 | N/A | 0.455 | 0.831 |
| **Insurance** (IKA/OAEE VS OPAD/other public) | 0.169 | **0.000** | 0.376 | 0.063 | 0.628 |
| **Disease duration** (long VS short) | 0.460 | 0.877 | 0.442 | 0.245 | **0.034** |
| **Disability status (EDSS)** (≤ 2.5 VS ≥ 3.0) | 0.293 | 0.653 | 0.648 | 0.520 | 0.087 |
| **Hospitalization** (yes VS no) | 0.573 | 0.172 | 0.982 | 0.086 | 0.429 |
| **Visit to one-day clinic** (yes VS no) | 0.622 | 0.346 | 0.705 | 0.685 | 0.547 |
| **Treatment duration** (long VS short) | 0.059 | 0.143 | 0.446 | 0.409 | **0.010** |

^a^The patients were categorized to those finding the received help/support excellent-very good or good-satisfactory and to those estimating the information as medium or insufficient.
