# Supplementary material for: Observational Study Assessing Demographic, Economic and Clinical Factors Associated with Access and Utilization of Health Care Services of Patients with Multiple Sclerosis under Treatment with Interferon Beta-1b (EXTAVIA)
Source: PLoS One. 2014 Nov 24;9(11):e113933. doi: 10.1371/journal.pone.0113933 (PMC4242657; doi:10.1371/journal.pone.0113933)
Supplement: Table S8 — Results of t-tests or one-way ANOVA for estimation of association of treatment omission times with baseline demographic and clinical characteristics of the treated population. (DOCX) [file pone.0113933.s008.docx]

| **Table S8:** Results of t-tests or one-way ANOVA for estimation of association of treatment omission times with baseline demographic and clinical characteristics of the treated population | | | |
| --- | --- | --- | --- |
| **Characteristic/subgroups** | **Treatment omission times (mean ± SD)** | **Statistic ^a^** | **p-value** |
| Age  old  young | 0.60 ± 1.04  0.58 ± 1.21 | 0.136 | 0.892 |
| Gender  male  female | 0.68 ± 1.12  0.56 ± 1.13 | 0.646 | 0.519 |
| Residence  urban centers  away from urban centers | 0.55 ± 1.11  0.68 ± 1.18 | 0.757 | 0.450 |
| Education  primary/no official  secondary  higher | 0.65 ± 1.21  0.70 ± 1.25  0.31 ± 0.65 | 2.147 | 0.120 |
| Employment status  working  not working (incl. retired) | 0.58 ± 1.14  0.61 ± 1.11 | -0.145 | .884 |
| Insurance  IKA/OAEE  OPAD/other public | 0.65 ± 1.17  0.52 ± 1.07 | 0.830 | 0.408 |
| Disease duration  long  short | 0.75 ± 1.24  0.44 ± 0.99 | 2.017 | **0.045** |
| Disability status (EDSS)  ≤ 2.5  ≥ 3.0 | 0.37 ± 0.81  0.88 ± 1.38 | -3.285 | **0.001** |
| Hospitalization  yes  no | 0.58 ± 1.00  0.61 ± 1.26 | 0.205 | 0.837 |
| Visit to one-day clinic  yes  no | 0.63 ± 0.99  0.57 ± 1.23 | -0.351 | 0.726 |
| Treatment duration  long  short | 0.81 ± 1.31  0.40 ± 0.88 | 2.636 | **0.009** |

^a^ t in case of t-test, F in case of one-way ANOVA
