# Supplementary material for: Observational Study Assessing Demographic, Economic and Clinical Factors Associated with Access and Utilization of Health Care Services of Patients with Multiple Sclerosis under Treatment with Interferon Beta-1b (EXTAVIA)
Source: PLoS One. 2014 Nov 24;9(11):e113933. doi: 10.1371/journal.pone.0113933 (PMC4242657; doi:10.1371/journal.pone.0113933)
Supplement: Table S9 — Results of Chi square tests for estimation of association of treatment omission with baseline demographic and clinical characteristics of the treated population. (DOCX) [file pone.0113933.s009.docx]

| **Table S9:**  Results of Chi square tests for estimation of association of treatment omission with baseline demographic and clinical characteristics of the treated population | | |
| --- | --- | --- |
|  | **Chi-square** | **p-value** |
| **Characteristic** |  |  |
| **Age** (old VS young) | 0.192 | 0.661 |
| **Gender** (male VS female) | 1.840 | 0.175 |
| **Residence** (urban centers VS away from urban centers) | 0.372 | 0.542 |
| **Education** (primary/no official VS secondary VS higher) | 2.349 | 0.309 |
| **Employment status** (working VS not working) | 0.103 | 0.748 |
| **Insurance** (IKA/OAEE VS OPAD/other public) | 0.036 | 0.849 |
| **Disease duration** (long VS short) | 7.252 | **0.007** |
| **Disability status (EDSS)** (≤ 2.5 VS ≥ 3.0) | 9.162 | **0.002** |
| **Hospitalization** (yes VS no) | 0.004 | 0.950 |
| **Visit to one-day clinic** (yes VS no) | 0.064 | 0.800 |
| **Treatment duration** (long VS short) | 8.311 | **0.004** |
